# Novel Flow Cytometric Immunoassay for Detection of Proinsulin Autoantibodies in Diabetes Mellitus Employing a Recombinant Autoantigen Expressed in *E. coli*

**DOI:** 10.3389/fimmu.2021.648021

**Published:** 2021-04-06

**Authors:** Adriana Victoria Sabljic, Silvina Sonia Bombicino, Juan Ignacio Marfía, Luciano Lucas Guerra, Alberto Penas Steinhardt, Natalia Inés Faccinetti, Rubén Francisco Iacono, Edgardo Poskus, Aldana Trabucchi, Silvina Noemí Valdez

**Affiliations:** ^1^ Universidad de Buenos Aires (UBA), Facultad de Farmacia y Bioquímica, Departamento de Microbiología, Inmunología, Biotecnología y Genética, Cátedra de Inmunología, Buenos Aires, Argentina; ^2^ Consejo Nacional de Investigaciones Científicas y Técnicas (CONICET) Universidad de Buenos Aires, Instituto de Estudios de la Inmunidad Humoral “Prof. Ricardo A. Margni” (IDEHU), Buenos Aires, Argentina; ^3^ Universidad Nacional de Lujan, Departamento de Ciencias Básicas, Laboratorio de Genómica Computacional, Buenos Aires, Argentina

**Keywords:** diabetes mellitus, autoimmunity, autoantibodies, proinsulin, flow cytometry, immunoassay

## Abstract

**Introduction:**

Insulin and proinsulin autoantibodies (IAA/PAA) are usually the first markers to appear in patients with type 1 Diabetes Mellitus (T1DM) and their prevalence ranges from 10 to 60% in the child-adolescent population. The reference method for IAA/PAA detection is the Radioligand Binding Assay (RBA), a highly specific and sensitive technique, but expensive and polluting. The aim of this work was to develop a novel flow cytometric microsphere-based immunoassay (FloCMIA) for PAA detection, employing recombinant human proinsulin (PI), as an alternative method to RBA, less expensive and harmful to the environment.

**Materials and Methods:**

Human PI was expressed as Thioredoxin fusion protein (TrxPI) in *E. coli* and a fraction was biotinylated. A double paratope model was used in which samples were incubated with TrxPI–biotin and microspheres adsorbed with TrxPI. The immune complexes were revealed using Streptavidin–Phycoerythrin. The geometric mean of the signals was analyzed, and the results were expressed as Standard Deviation scores (SDs). Sera from 100 normal human control and from 111 type 1 diabetic patients were evaluated by FloCMIA. To correlate the novel assay with RBA, 51 diabetic patients were selected, spanning a wide range of PAA reactivity by RBA.

**Results:**

The study of ROC curves allowed choosing a cut-off value of 3.0 SDs and the AUC was 0.705, indicating that FloCMIA has fair ability to distinguish between samples from each group. A prevalence of 50% for PAA was obtained in the population of diabetic patients studied. The specificity was 96% and the analytical sensitivity (percentage of patients RBA positive, also positive by FloCMIA) was 69%. There was a substantial agreement between methods (kappa statistic=0.700).

**Conclusions:**

A novel immunoassay based on flow cytometry that uses easy-to produce recombinant PI was developed. This assay constitutes an innovative and cost-effective alternative to RBA for the determination of PAA in patients’ sera. The method developed here, presents good performance and a wide dynamic range together with a small required sample volume. Furthermore, these results make it possible to develop multiplex immunoassays that allow the combined detection of autoantibodies present in T1DM and other related autoimmune diseases.

## Introduction

Type 1 Diabetes Mellitus (T1DM) is an autoimmune disorder originated by the loss of tolerance towards components of insulin-producing pancreatic beta cells in genetically predisposed subjects. The destruction of beta cells is mediated by cytotoxic T lymphocytes and, due to this damage, several pancreatic autoantigens are released into the bloodstream and different autoantibodies are produced. The four main autoantibodies associated with risk of progression to clinical disease are: insulin/proinsulin autoantibodies (IAA/PAA), glutamic acid decarboxylase autoantibodies (GADA), insulinoma associated protein tyrosine phosphatase 2 autoantibodies (IA-2A) and zinc transporter isoform 8 autoantibodies (ZnT8A) (1–3). These markers can be detected in blood circulation long before the clinical symptoms are manifested (4) and, as this pathology is characterized by an asymptomatic period that can last within few months to more than ten years (5), the measurement of these autoantibodies can be very useful as risk predictors of T1DM in children and young adults (6).

IAA/PAA are often the first autoantibodies to appear during the natural history of the disease (7, 8) and their titer is remarkably high in young children, with an inverse correlation between their presence and the age of onset of the disease (9). Therefore, the prevalence of IAA/PAA varies widely according to patients’ age range (50–60% in subjects younger than 10 years and around 10% in post pubertal ones) (10) and regarding its ethnic origin (11).

The method historically considered as the reference one for IAA/PAA detection is the Radioligand Binding Assay (RBA) (12–15). This technique is widely used due its high specificity and sensitivity; however, as it uses radioactive tracers, is harmful to the environment, presents an elevated cost and is restricted only to authorized laboratories. Therefore, it is imperative to have alternative methods to the RBA and, in this sense, several research groups have worked on their development. Unlike what happens for the other markers of T1DM, until now it was not possible to develop an enzyme-linked immunosorbent assay (ELISA) with good performance for IAA/PAA detection. This may be due, in part, to the possible denaturation of the antigen (insulin/proinsulin) when it is immobilized on the solid phase and, besides, because of its low molecular weight critical epitopes may be hidden during immobilization, making them inaccessible to autoantibodies, contrary to what happens in fluid phase immunoassays (16). In RBA, antigen-antibody interaction occurs in a high dilution in the liquid phase, almost reaching the equilibrium state. Instead, in ELISA this interaction occurs in a solid phase where the amount of immunocomplexes formed is highly dependent on antibodies concentration. Taking this into account, and due to the really low concentration of autoantibodies in type 1 diabetic patients’ sera (in the order of 10^-12^ M), their detection employing solid phase assays is a real challenge. Likewise, multiple international workshops (Diabetes Antibody Standardization Program 2000, 2002, 2003 and 2005) (17) have reported the difficulty among different laboratories to achieve immunoassays with good performances for the detection of this marker. Over the last few years, the detection of these autoantibodies based on electrochemiluminescence has shown to achieve similar, and even better, performances than the RBA (18, 19). Nevertheless, this method requires MSD (Meso Scale Discovery) electrochemiluminescent instrument that is not available in mostly all medium complexity laboratories and hospitals of our country.

We have previously described the production of recombinant human proinsulin (PI) in *Escherichia coli (E. coli)* as a soluble and properly folded fusion protein with Thioredoxin (Trx). Also, it was possible to improve its yielding by recovering the chimera from inclusion bodies. This chimera retains full immunochemical reactivity (20) and can be easily produced and purified. Having a handy source of native PI, allowed us to develop a highly sensitive and quasi-quantitative protocol of flow cytometric microsphere-based immunoassay (FloCMIA) for PAA determination.

In this sense, we have previously designed alternative FloCMIA protocols for the detection of GADA, obtaining the best performance with the one based on the simultaneous interaction of GADA with TrxGAD65 immobilized on the surface of the microsphere and with TrxGAD65–biotin in the fluid phase (21).

So, the aim of the present work was to develop a FloCMIA protocol for the detection of PAA employing PI produced in *E. coli*, as an alternative method to RBA, less expensive and harmful to the environment.

## Materials and Methods

### Human Sera Collection

Serum samples were obtained from fasted individuals (8 hours) and then, stored at -20°C until assayed. The groups under study were:

Normal Human Control. Normal control sera (*n* = 100, mean age of 32 ± 8 years with median age of 30.5, range 21 to 60 and male/female: 38/62) were obtained from healthy subjects without personal or family history of Diabetes Mellitus or other autoimmune diseases. These sera were employed to calculate the cut-off value of the immunoassays. The collection of serum samples was approved by the Ethics Committee of the Clinical Hospital José de San Martín, University of Buenos Aires, Buenos Aires, Argentina. All subjects were informed about the purpose of the study, and a signed consent for study participation was obtained.

Type 1 Diabetic Patients. Serum samples (*n* = 111) were collected from children and adolescents admitted to the Nutrition Service at Gutierrez National Pediatric Hospital, Buenos Aires, Argentina, recently diagnosed according to WHO criteria (22). Samples were taken before or within 72 h of starting insulin treatment with the approval of the corresponding Ethical Committee and parental consent was obtained. In these samples, the other autoimmune markers GADA, IA-2A and ZnT8A, were determined by RBA (**Supplementary Figure 1**). To correlate the performance of FloCMIA with that of the reference method, out of these 111 samples, 51 were selected, spanning a wide range of PAA reactivity by RBA (mean age of 7.1 years at diagnosis, with median age of 8.0, range 0.1–6 years and male/female: 23/28).

### PAA Detection by Radioligand Binding Assay

RBA for PAA was performed as previously described by our group (15). Briefly, following manufacturer instructions, cDNA coding for human PI was transcribed and translated using a rabbit reticulocyte lysate system in the presence of [^35^S]-cysteine (New England Nuclear, Boston, MA, USA). [^35^S]-PI was refolded by a disulfide reduction-reoxidation procedure, and finally, isolated by reverse phase HPLC.

Thirty microliters of sera were incubated with 1000 cpm of [^35^S]-PI in 90 μl of RBA buffer (50 mM sodium phosphate, 100 mM NaCl, pH 7, 0.1% Aprotinin and 0.1% bovine serum albumin -BSA-) for seven days at 4°C. Afterwards, to isolate the immunocomplexes, 50 μl of a 50% suspension of Protein G - Sepharose 4B FF (Amersham Biosciences, Piscataway, NJ, USA) in RBA buffer were added and the mixture incubated for 2 h at room temperature on an end-over-end shaker. Samples were centrifuged and supernatants were discarded. Pellets were washed four times with 200 μl of RBA buffer, and suspended in 100 μl of 1% sodium dodecyl sulphate (SDS). After a centrifugation of 5 minutes at 6000×*g*, supernatants were carefully transferred to appropriate vials for scintillation counting (5 min per tube). Results were calculated as Bound percentage (B%):

$$B\%=100\times \frac{bound\text{ cpm}}{total\text{ cpm}}$$

and expressed as Standard Deviation scores (SDs):

$$SDs=\frac{\left(B\%-Bc\%\right)}{SDc}$$

where Bc% was the mean B% of control sera and SDc its standard deviation. An assay was considered positive if SD score >3.0. Thirty normal human sera were included as controls and Bc% was normally distributed. As we have previously reported (15), the sensitivity of RBA using ^35^S-PI was 63.4%.

### Expression of Proinsulin as a Recombinant Protein in *E. coli*, Purification and Biotinylation

#### Proinsulin Expression and *E. coli* Disruption

PI was expressed as a fusion protein with Trx (TrxPI) in *E. coli* as previously described (20). Briefly, *E. coli* strain GI724 was transformed with the recombinant vector pTrxPI. Bacteria were cultured in a medium containing 0.2% casein amino acids, 0.5% glucose, 1 mM MgCl_2_ and 100 μg/mL ampicillin at 30°C. Protein expression was induced with 100 μg/mL Tryptophan for 3 h at 37°C. In order to obtain total cell lysate, bacteria from 200 mL of culture were collected by centrifugation. Following a freezing and thawing cycle, the pellet was resuspended in 4 mL of lysis buffer (50 mM Tris–HCl, 100 mM NaCl, 1 mM EDTA, pH 7.0) and then sonicated in the presence of 1 mM 2-mercaptoethanol (2ME) and protease inhibitors (0.1% v/v aprotinin and 2 mM phenylmethylsulphonyl fluoride -PMSF-) over crushed ice. After sonication, Triton X- 100 was added to a final concentration of 0.1%, and the mixture was incubated for 10 min at 0°C. The intracellular soluble fraction (ISF) was separated from the inclusion bodies (IB) by centrifugation at 15000×*g* for 10 min at 4°C.

#### Protein Purification from ISF by Affinity Chromatography

TrxPI was purified from the ISF by affinity chromatography following the protocol previously described with minor modifications (20). The resin was based on an agarose support covalently modified with phenylarsine oxide, which permitted the binding of proteins containing vicinal dithiol residues as it occurs in Trx (23). ISF (≈ 4 mL) was added to the resin, previously equilibrated with lysis buffer, and activated with lysis buffer containing 20 mM 2ME; the resulting suspension was incubated for 1.5 h at 4°C. The resin was washed sequentially using lysis buffer with increasing concentrations of 2ME and, finally, the bounded proteins were eluted with several 2 mL aliquots of lysis buffer containing 100 mM 2ME. The protein concentration in purified fractions was determined using the Coomasie Plus (Bradford) Assay kit (Thermo Fisher Scientific, Waltham, MA, USA).

#### Protein Biotinylation

Two milliliters of the purified recombinant protein were subjected to buffer exchange to Phosphate Buffered Saline (PBS: 1.5 mM KH_2_PO_4_, 8.1 mM Na_2_HPO_4_, 140 mM NaCl, 2.7 mM KCl, pH 7.4) using a PD-10 desalting column (GE Healthcare, Chicago, IL, USA) according to the manufacturer’s instructions. The desalted protein was then incubated for 2 h at 0°C with 20-fold molar excess of sulfo-NHS-biotin (Pierce Biotechnology, Rockford, IL, USA) and free biotin was removed on a new PD-10 desalting column. The biotinylated protein was stored in a mixture containing 50% v/v glycerol, 50 mM 2ME and 0.1% w/v aprotinin.

#### Protein Isolation From IB

On the other hand, TrxPI was isolated from the IB by a first step of solubilization with 8 M urea in 0.1 M Tris, pH 8.5 followed by an oxidative refolding process through dialysis at 4°C against 0.5 M L-arginine, 50 mM Tris–HCl, 5 mM EDTA, 5 mM reduced glutathione and 0.5 mM oxidized glutathione, pH 9.5 (24). The refolded protein´s buffer was exchanged to PBS using a PD-10 desalting column and the protein was stored in fractions with 50 mM 2ME and 0.1% w/v aprotinin.

#### Sodium Dodecyl Sulphate-Polyacrylamide Gel Electrophoresis and Western Blot Analysis

Fractions at different steps of purification of TrxPI were collected, diluted in 0.2 mL of SDS–polyacrylamide gel electrophoresis (SDS-PAGE) sample buffer (50 mM Tris–HCl, 12.0% glycerol, 0.005% bromophenol blue, 4.0% SDS, 4.0% 2ME, pH 6.8) and boiled for 5 minutes for SDS-PAGE and western blotting (WB) analysis. For comparison, all SDS-PAGE lanes in each gel contained proteins recovered from the same number of cells. For WB, bands were transferred to nitrocellulose membranes. Unoccupied binding sites in the membrane were blocked with 3% skim milk in Tris buffer saline (TBS: 50 mM Tris–HCl, 150 mM NaCl, pH 7.5) by overnight incubation at 4°C. After three washing steps with TBS added with 0.05% Tween 20 (TBS-T), the membrane was incubated for 2 h at room temperature with purified polyclonal serum to Trx diluted 1:100 in 3% w/v BSA TBS-T and then washed five times with TBS-T. Bound antibodies were visualized by incubation with peroxidase-conjugated goat antibodies to rabbit IgG (Jackson ImmunoResearch Laboratories, Inc., West Grove, PA, USA) diluted 1/1500 in 3% w/v BSA–TBS-T followed by the addition of α-chloronaphthol (Sigma-Aldrich, Inc., St. Louis, MO, USA) and 10 vol H_2_O_2_.

### PAA Detection by Flow Cytometric Microsphere-based Immunoassay (FloCMIA)

#### Passive Adsorption of Microspheres With TrxPI

This protocol was performed using the manufacturer’s instructions (Spherotech Inc., Lake Forest, IL, USA) with minor modifications. Thirty-three microliters of a suspension of 5 μm microspheres 5% w/v were mixed in a 0.5 mL Eppendorf tube with 30 μg TrxPI isolated from IB per 100 cm^2^ of microspheres in a final volume of 220 μL in Isotonic Buffered Saline (IBS: 2.3 mM NaH_2_PO_4_, 14.2 mM Na_2_HPO_4_, 140 mM NaCl, 3.8 mM KCl, pH 7.4) and incubated overnight at 4°C on an end-over-end shaker. The preparation was centrifuged at 12000×*g* for 10 min and the supernatant was discarded. In order to block microsphere’s free sites, the pellet was suspended with 220 μL of IBS-BSA 0.5% w/v and incubated for 1 h at room temperature on an end-over-end shaker. The preparation was centrifuged again at 12000×*g* for 10 min, the supernatant was discarded, and the pellet was washed with 400 μL of PBS. Finally, another 400 μL of PBS were added, resulting in 5x10^4^ microspheres–TrxPI/μL and the suspension was stored at 4°C.

#### FloCMIA Protocol

The immunoassay was performed as previously described (21) with minor modifications. Except when otherwise indicated, all washing steps were performed with 200 μL of PBS-Tween 20 0.05% v/v (PBS–T) on a Multiscreen_HTS_ vacuum manifold (Millipore Corporation, Billerica, MA, USA). Reagent dilutions were prepared using 0.5% w/v BSA in PBS–T.

**Figure 1:**
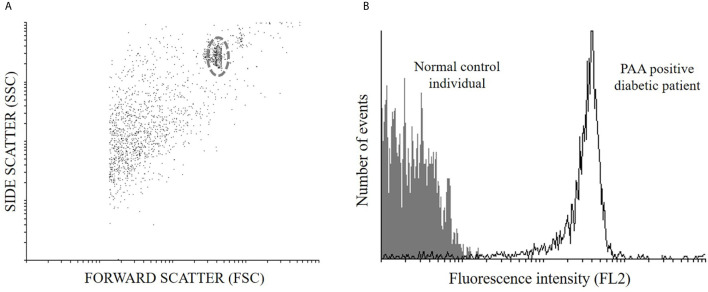
Signal processing. **(A)** Population of 5 μm microspheres adsorbed with TrxPI observed in a Dot plot graph of side scatter (SSC) vs. forward scatter (FSC) for purposes of gating **(B)** Histogram of number of events vs. Fluorescence intensity (FL2 channel) to measure the fluorescence associated with each sample; representative signals are shown for the sera from a normal control individual and a PAA positive diabetic patient.

Twenty microliters of pure human serum were mixed with 20 μL of a microsphere–TrxPI preparation (3.3x10^2^ microspheres/μL) and with 3.6 ng of TrxPI–biotin in a final volume of 60 μL in 0.5 mL Eppendorf tubes. Incubation was performed in 2 steps: (i) 2 h at room temperature and (ii) 5 days at 4°C, both on an end-over-end shaker. After the incubation steps, mixtures were transferred to a Multiscreen_HTS_-HV 96-well filtration plate (Millipore Ireland BV) and washed 5 times. For detection of the bound antibody, 6.25 ng of Streptavidin-Phycoerythrin (MiltenyiBiotec, Germany) in a final volume of 50 μL were added in each well and incubated for 1 h at room temperature in the dark on a shaker. After that, plates were washed 4 times plus 1 final washing step with 200 μL of PBS and microspheres were suspended with another 200 μL of PBS. The suspension was transferred to Röhren tubes (3.5 mL, 55 x 12 mm, Sarstedt, Germany) and acquired on a PAS III PARTEC flow cytometer (PARTEC, Görlitz, Germany) or a Becton Dickinson FACSCalibur flow cytometer (Franklin Lakes, NJ, USA) equipped with a 488 nm argon laser. The data obtained for each sample were analyzed using Cyflogic software (CyFlo Ltd., Turku, Finland), identifying, firstly, the singlet population of microspheres by gating in a forward scatter (FSC) vs. side scatter (SSC) dot plot. Secondly, fluorescence signals were measured in an FL2 channel and reported as the Geometric Media of Fluorescence Intensity (GeoM) (**Figure 1**). Results were expressed as SDs:

$$SDs=\left(\frac{GeoMm-GeoMc}{SDc}\right)$$

where GeoMm is the mean GeoM of samples in duplicate, GeoMc is the mean GeoM of normal human control sera and SDc is its standard deviation.

Twenty-five human control sera were included in each assay and samples were considered positive when SDs >3.0.

### Statistical Analysis

The normal distribution of the data was analyzed by the D’Agostino & Pearson Omnibus normality test. To select the cut-off with maximum sensitivity and specificity, ROC curves were made by plotting these parameters against the corresponding cut-off values. Statistical significance was assessed by parametric tests: Student’s t-test for unpaired samples with Welch correction; or non-parametric tests: Mann-Whitney U test for unpaired data, when applicable. Spearman coefficient of correlation (r_s_) was calculated to evaluate inter-assay correlation. The degree of agreement between RBA and FloCMIA was evaluated by calculating the kappa statistic. A kappa value of 0.01–0.20 was indicative of slight agreement; 0.21–0.40, fair agreement; 0.41-0.60, moderate agreement; 0.61–0.80, substantial agreement; and 0.81–1.00, almost perfect or perfect agreement (25–28). Calculations were performed using GraphPad Prism version 6.01 for Windows (GraphPad Software, San Diego California, USA, www.graphpad.com). A value of p <0.05 was considered statistically significant.

## Results

### Expression of Proinsulin as a Recombinant Protein in *E. coli*, Purification and Biotinylation

**Figure 2:**
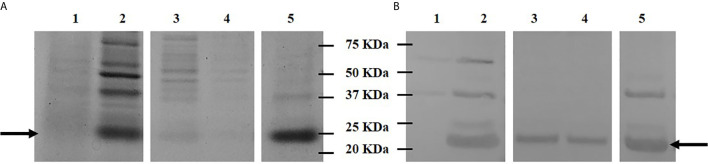
Purification of TrxPI from ISF by affinity chromatography and isolation from IB. Analysis of TrxPI fractions by **(A)** SDS-PAGE (12.1 % T, 6.0 % C, 1 mm, under reducing conditions, stained with Coomassie Brilliant Blue R-250) and **(B)** WB revealed with a rabbit polyclonal serum to Thioredoxin as primary antibody. In **(A, B)**, lane 1 corresponds to total lysate of transformed *E coli* strain GI724 before induction, lane 2: total lysate of transformed *E coli* strain GI724 after induction, lane 3: purified TrxPI from ISF, lane 4: TrxPI biotinylated, lane 5: TrxPI from IB. Arrows indicate the electrophoretic mobility of TrxPI.

As previously described, recombinant fusion protein TrxPI was efficiently expressed in *E. coli* strain GI724 after 3 h induction. The ISF was analyzed by SDS-PAGE showing one band with the expected molecular weight (≈ 22 kDa) for the full-length engineered protein in the fractions obtained before and after purification (**Figure 2A**). A typical TrxPI preparation yielded ≈ 6.00 mg of TrxPI/L of culture medium. The identity of TrxPI was confirmed by WB analysis using a specific and purified rabbit polyclonal serum against Trx (**Figure 2B**).

TrxPI isolated from the IB was also analyzed with SDS-PAGE and WB and, as it is shown in **Figure 2**, there was a band consistent with the expected molecular weight of the recombinant fusion protein that was also recognized by the purified anti Trx serum. Final yield of properly refolded 80–85% pure TrxPI isolated from de IB was 9 mg/L bacterial culture.

### PAA Detection by Flow Cytometric Microsphere-Based Immunoassay (FloCMIA)

**Figure 3:**
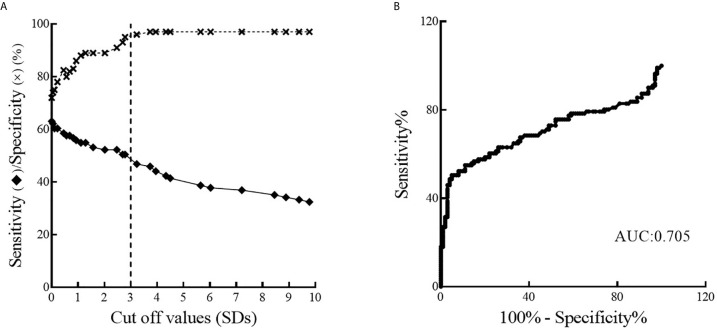
Analysis of the performance of FloCMIA resulting from the study of 100 sera from normal control individuals and 111 sera from infant-juvenile patients with type 1 Diabetes. **(A)** Sensitivity curve (solid line) and specificity (broken line) as function of the possible cut-off values. The vertical dashed line indicates the cut-off value with the optimized sensitivity and specificity parameters (cut-off = 3.0). **(B)** ROC curve analysis of FloCMIA, AUC is included.

One hundred human control sera and 111 type 1 diabetic patients’ sera were analyzed by FloCMIA to determine the presence of PAA. ROC curves were performed to select the cut-off value with maximum sensitivity and specificity (**Figure 3**); the chosen one was 3.0 SDs. Moreover, the area under the ROC curve (AUC) was 0.705, indicating that the method has a fair ability to distinguish between samples from the two groups under study (29). A rabbit policlonal serum to proinsulin was used as the assay internal standard positive control. In order to calculate the coefficient variation a serum from a PAA positive patient was employed. The intra-assay coefficient variation was 1.73% (n = 4) and the inter-assay coefficient variation was 10.72% (n = 5).

**Figure 4:**
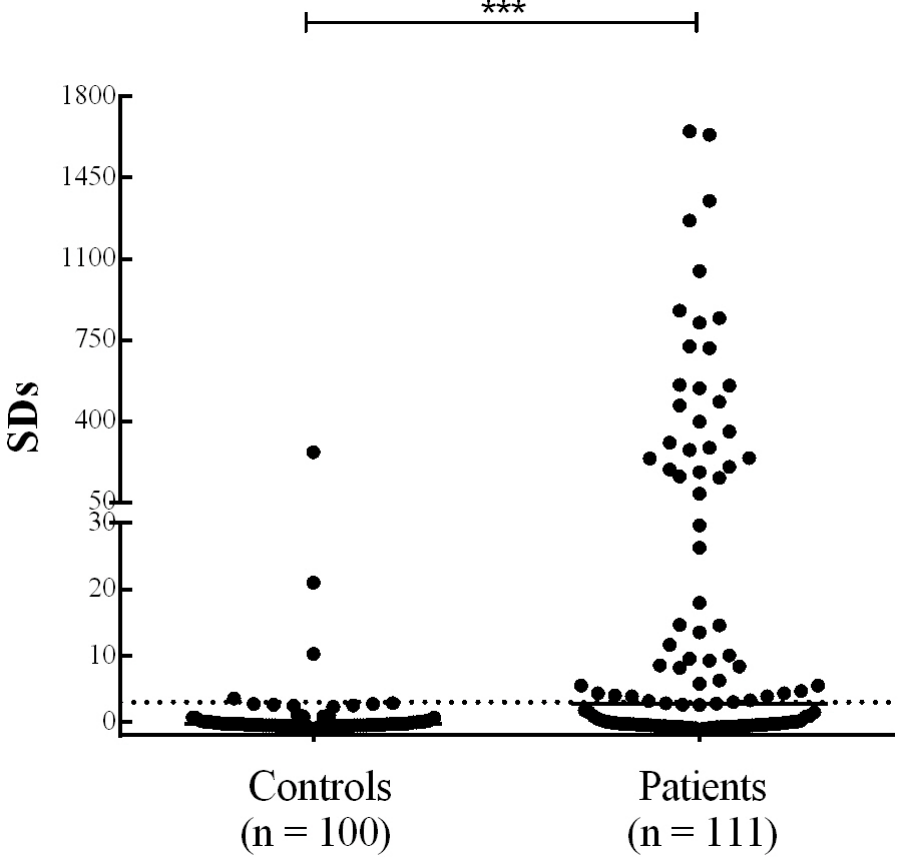
PAA results obtained by FloCMIA in sera from normal control individuals and sera from type 1 diabetic patients. Results are expressed as SDs. The cut-off value (SDs >3.0) is indicated by a dotted line and medians for each population are indicated as a full line (***p < 0.0001).

The results obtained, expressed as SDs (range: -1.11 to 1650), are shown in **Figure 4** and the Raw data is shown in **Supplementary Table 1**. Out of 111 type 1 diabetic patients analyzed, 55 (Sensitivity: 50%) were positive by FloCMIA. The specificity, calculated as 100 % minus the percentage of normal human sera detected as positive, was 96.0%. The median SDs of normal human controls (- 0.33) was significantly different from that of the patients (3.01), p< 0.0001. The clinical characteristics of the two subsets of diabetic patients positive or negative by FloCMIA, were summarized in the **Table 1**. No significant differences were found between both groups for any of the characteristics studied.

**Table 1:** Clinical characteristics for two subsets of diabetic patients positive (n=55) or negative (n=56) by FloCMIA.

		FloCMIA +	FloCMIA -	p-value
*n*		55	56	–
Mean age at debut (years)		7.9 ± 4.3	8.6 ± 4.8	0.485
Age range (years)		0.6 - 16	0.05 – 18	–
Male/Female		28/27	26/30	0.501
Ketoacidosis at debut		25	23	0.504
Family history of T1DM		8	7	0.636
Family history of T2DM		27	26	0.679
Other autoimmune disorders		2	1	0.300
BMI (kg/m^2^)	<18.5	3	8	0.015
	18.5-24.9	44	36	
	25.0-29.9	6	4	
	>30.0	2	8	
Other markers of T1DM positive by RBA		43	42	0.578

### Comparison of PAA detection by FloCMIA Using Recombinant TrxPI Produced in Bacteria and by RBA Using *In Vitro* Translated ^35^S-PI

For further evaluation of the ability of FloCMIA to detect PAA, 51 sera from type 1 diabetic patients that were positive by RBA, and 100 samples from normal human control sera were analyzed.

**Figure 5:**
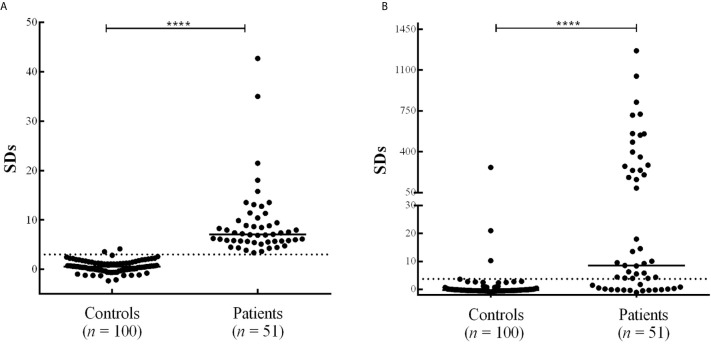
PAA results obtained by RBA **(A)** and FloCMIA **(B)** from 100 normal human sera (controls) and 51 type 1 diabetic patients’ sera. Results are expressed as SDs. The cut-off value (SDs >3.0) is indicated by a dotted line and medians for each population are indicated as a full line (****p < 0.0001).

The antibodies levels employing RBA, expressed as SDs (range: -2.36 to 42.69) are shown in **Figure 5A**. The signals obtained from type 1 diabetic patients were characterized by a median of 7.07 and a mean of 9.18. On the other hand, the parameters observed for the normal human control sera were a median of 0.55 and a mean of 0.57. Furthermore, the specificity was 98% (**Table 2**).

**Table 2:** Analytical parameters of RBA and FloCMIA from normal human controls and type 1 diabetic patients.

	RBA		FloCMIA	
	Type 1 diabetic patients	Normal controls	Type 1 diabetic patients	Normal controls
*n*	51	100	51	100
Mean (SDs)	9.18	0.57	181.77	0.00
Median (SDs)	7.07	0.55	8.53	-0.33
Range (SDs)	(-2.36) – 42.69		(-1.11) – 1265.31	
Cut-off (SDs)	3.0		3.0	
Analytical Sensitivity^*^(%)	–		69	
Specificity^†^ (%)	98		96	

^*^Percentage of patients RBA positive that were also positive by FloCMIA.

^†^100 minus the percentage of false positives.

As it is shown in **Figure 5B**, FloCMIA had a wide range of SDs signals (from -1.11 to 1265.31). Besides, out of the 51 type 1 diabetic patients PAA positive by RBA evaluated, 35 scored positive by this novel assay, obtaining an Analytical Sensitivity of 69% (calculated as the percentage of patients RBA positive that were also positive by FloCMIA). This population was characterized by a median of 8.53 and a mean of 181.77. The specificity obtained was 96% and the median SDs of normal human controls (-0.33) was significantly different from that of the patients (8.53), p< 0.0001 (**Table 2**).

### Integrated Results and Correlation Analysis

**Figure 6:**
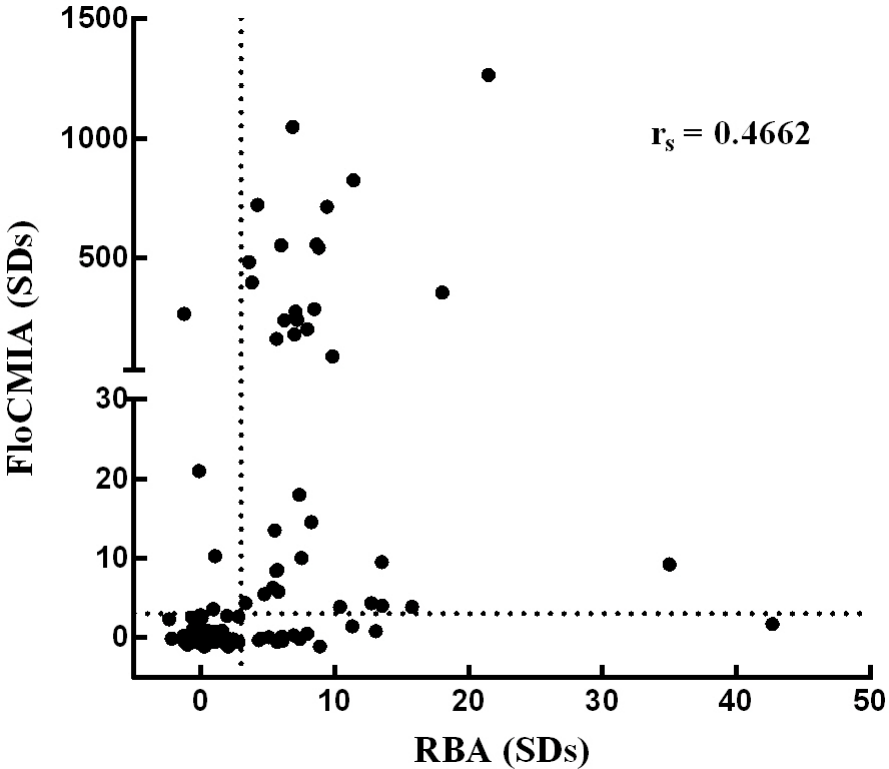
Comparison of PAA detection by FloCMIA using recombinant TrxPI produced in bacteria and by RBA using *in vitro* translated ^35^S-human PI. One hundred sera from normal control individuals and from 51 type 1 diabetic patients were tested. Results are expressed as SDs. Values above cut-off dotted lines were considered positive.

**Figure 6** shows a statistical comparison of RBA and FloCMIA results from 100 normal human sera and 51 type 1 diabetic patients’ sera. Despite the differences in the physical–chemical principles of both compared techniques, a significant correlation was found between the levels of PAA measured by RBA and by FloCMIA (Spearman’s coefficient, r_s_=0.4662; p<0.0001). Moreover, the concordance between the two immunoassays was 96.7%, with a kappa statistic of 0.700, representing a substantial agreement between the two methods. Thus, the correlation between RBA and FloCMIA for the determination of PAA was real and not by random sampling.

## Discussion

Diabetes Mellitus is currently one of the main social health problems due to its prevalence, its specific complications and the presence of other pathologies that are usually linked with it. T1DM is a relatively common disorder that develops in genetically susceptible individuals and is associated with anti-islet autoantibodies, which can be found years before the clinical onset of the pathology. A series of well-characterized assays are currently available for the detection of the involved autoantibodies, evaluated through international quality control workshops, which are of utmost importance both for the prediction and diagnosis of T1DM, as well as for the design of trials for disease prevention.

In children followed prospectively from birth, IAA/PAA are frequently the first autoantibodies to appear before the clinical onset of T1DM (30). The methods to assess the presence of IAA/PAA are validated through workshops of the Diabetes Autoantibody Standardization Program (DASP) or, nowadays, the Islet Autoantibody Standardization Program (IASP) from the Center for Disease Control and Prevention (CDC) and the Immunology of Diabetes Society (17, 31). These workshops demonstrated that despite the ELISA assays are able to detect anti-insulin antibodies after subcutaneous injections of human insulin, the standard ELISA formats were not capable of detecting IAA present in recent onset diabetic patients or in individuals progressing to T1DM (16, 32, 33). These ELISA assays use immobilize insulin in the plate wells so there is a high chance that some essential insulin epitopes may be hidden.

Taking into account that there is evidence that describes that all IAA-positive patients react with PI in fluid phase radiometric assays (15, 33) and given the need to improve the assays for the detection of these autoantibodies, the TrxPI chimera was designed. The purpose of this design was to preserve the critical determinants in the specific recognition by IAA/PAA and, in addition, it is known that IAA/PAA appear in circulation as a consequence of the damage suffered by pancreatic beta cells, with the subsequent release of the precursors of insulin with autoantigenic potency stored in these cells. Therefore, the use of proinsulin representing the complete precursor of the insulin molecule could be an appropriate alternative for the measurement of IAA/PAA in sera from patients with autoimmune diabetes, allowing the detection of antibodies against insulin and C peptide.

Despite the good performance of RBA in the diagnosis of autoimmune diabetes, it is imperative to develop alternative non-radiometric assays since the use of radioactive materials is falling into disuse due to the multiple disadvantages that they present (high cost, environmental impact, low applicability in medium and low complexity laboratories, centralization of determinations, etc.). For this reason, some authors have developed autoantibody assays that do not require radioactivity. Yu et al. have described a highly sensitive and specific electrochemiluminescence assay for IAA determination (18). But since this assay requires an Imager SQ120 plate readers (MSD) which is not available in most laboratories and public hospitals in our country, we proposed a method that requires a flow cytometer, an equipment commonly used for the analysis and characterization of cell populations.

In this sense, human proinsulin fused to thioredoxin was expressed in *E. coli* as previously described (20), yielding approximately 6 mg of TrxPI/L culture medium from intracellular soluble fraction and 9 mg of 80–85% pure properly refolded recombinant protein isolated from inclusion bodies. This chimera was then used to develop a flow cytometric microsphere-based immunoassay for the detection of PAA in type 1 diabetic patients’ sera. For this purpose, microspheres were coated with TrxPI from IB and, on the other hand, TrxPI from ISF was biotinylated. Both reagents were then incubated with human sera as described in the Materials and Methods section.

To evaluate the FloCMIA performance, 111 sera from patients with recent diagnosis of T1DM according to clinical criteria and 100 sera from normal human controls were analyzed. The sensitivity, calculated as the percentage of patients that scored positive, was 50%, and the specificity, calculated as 100 minus the percentage of normal human sera detected as positive, was 96%. These results agree with those reported by other groups (11, 34–38).

For further evaluation of the novel assay, 51 sera from PAA-positive type 1 diabetic patients, previously assayed by RBA, were analyzed by FloCMIA, reaching 69% of Analytical Sensitivity regarding the radiometric method, with a wide range of SDs signals (from -1.11 to 1265.31 and a median of 8.53 as shown in **Figure 5**). Moreover, there was a substantial agreement between them as defined by the statistic kappa, demonstrating that the correlation between RBA and FloCMIA for PAA determination was real and not by random sampling.

In conclusion the microsphere-based immunoassay performed on Flow cytometers constitutes an innovative and cost-effective alternative to traditional determination of PAA by RBA and/or ECL. Its advantages can be listed as follows: (i) highly sensitive detection due to Fluorescent-labeling, (ii) the use of easy-to produce recombinant proinsulin from prokaryotic cells, (iii) intact immunoreactivity of the antigen after adsorption on microspheres, (iv) the combination of solid and fluid phase interactions, (v) a good specificity, (vi) a wide dynamic range, (vii) a small sample volume required, (viii) low cost and environmentally harmless, (ix) applicable in most medium-complexity laboratories and public hospitals, as it does not require special equipment or authorization by the Nuclear Regulatory Authority and (x) it does not require acid treatment of the samples, as with electrochemiluminescence-based assay, making it a simpler execution method.

Likewise, the results obtained for PAA detection by FloCMIA make it possible to develop multiplex immunoassays that allow the combined detection of autoantibodies present in T1DM and other related autoimmune diseases such as autoimmune thyroid disease and celiac disease, through the use of microspheres of different size or internal fluorescence easily distinguishable on the flow cytometer. The possibility of detecting multiple markers differentially in a single analytical act and with a small sample volume, has a direct impact on the requesting physicians, not only because they can know the autoimmunity profile of the patient with a single analytical determination, but also because it is possible to request the analysis of the combinatorial of markers appropriate according to the clinic and age of each patient.

## Data Availability Statement

The raw data supporting the conclusions of this article will be made available by the authors, without undue reservation.

## Ethics Statement

The studies involving human participants were reviewed and approved by Ethics Committee of the Clinical Hospital José de San Martín, University of Buenos Aires, Buenos Aires, Argentina and Ethics Committee of the Nutrition Service at Gutierrez National Pediatric Hospital, Buenos Aires, Argentina. Written informed consent to participate in this study was provided by the participants’ legal guardian/next of kin.

## Author Contributions

AS, SB and SV conceived and designed the experiments. AS and SB performed the experiments. AS, SB and SV analyzed and interpreted the data. AT, RI, EP and SV contributed reagents/materials/analysis tools. AS, SB, AT and SV wrote the paper. AT, JM, LG, APS and NF contributed with the performance of experiments. EP, AT and SV supervised the research. All authors contributed to the article and approved the submitted version.

## Funding

This work was supported in part by grants from FONCYT Program of the National Agency for Science and Technology Promotion (ANPCYT) PICT-2014-1928 and PICT-2017-1436, National Research Council (CONICET) PIP 11220120100256CO and PUE 22920160100017CO and the University of Buenos Aires (UBA 20020150100115BA).

## Conflict of Interest

The authors declare that the research was conducted in the absence of any commercial or financial relationships that could be construed as a potential conflict of interest.

## Abbreviations

2ME, 2-mercaptoethanol; B%, Bound percentage; BSA, Bovine serum albumin; ELISA, Enzyme-linked immunosorbent assay; FloCMIA, Flow Cytometric Microsphere-based Immunoassay; GADA, Glutamic acid decarboxylase autoantibodies; GeoM, Geometric Media of Fluorescence Intensity; IA-2A, Insulinoma associated protein tyrosine phosphatase 2 autoantibodies; IAA/PAA, Insulin/Proinsulin autoantibodies; IB, Inclusion bodies; IBS, Isotonic Buffered Saline; ISF, Intracellular soluble fraction; PBS, Phosphate Buffered Saline; PBS-T, PBS with Tween 20 0.05% v/v; PI, Proinsulin; RBA, Radioligand Binding Assay; SDS, Sodium Dodecyl Sulphate; SDs, Standard Deviation scores; SDS-PAGE, SDS-polyacrylamide gel electrophoresis; T1DM, Type 1 Diabetes Mellitus; TBS, Tris buffer saline; TBS-T, TBS with 0.05% Tween 20; Trx, Thioredoxin; TrxPI, PI as a fusion protein with Thioredoxin; WB, Western blot; ZnT8A, Zinc transporter isoform 8 autoantibodies.
