# Supplementary material for: Novel Flow Cytometric Immunoassay for Detection of Proinsulin Autoantibodies in Diabetes Mellitus Employing a Recombinant Autoantigen Expressed in E. coli
Source: Front Immunol. 2021 Apr 6;12:648021. doi: 10.3389/fimmu.2021.648021 (PMC8056981; doi:10.3389/fimmu.2021.648021)
Supplement: Supplementary file 1 [file DataSheet_1.docx]

Supplementary Material

## Supplementary Figures


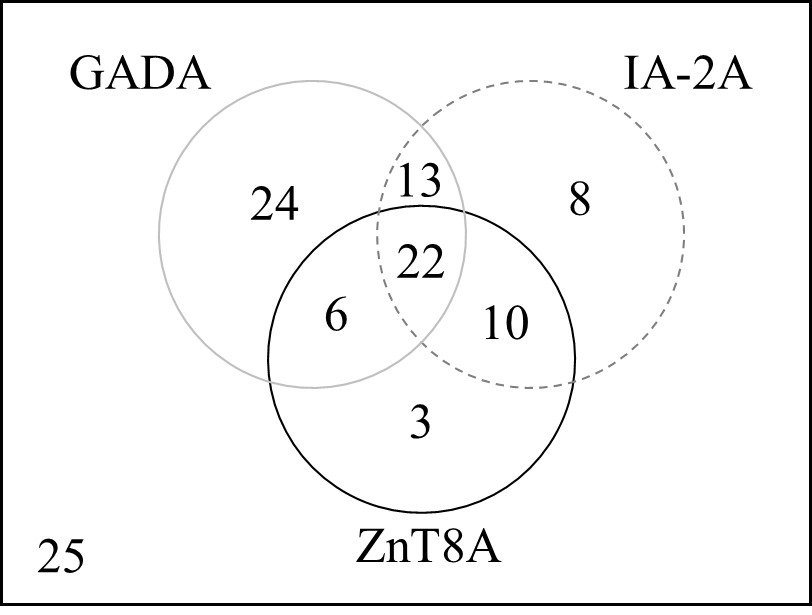


**Supplementary Figure 1: GADA, IA-2A and ZnT8A determination by RBA**. The results obtained for the three markers in the 111 type 1 diabetic patients’ sera by RBA are presented as a Venn diagram. The regions of intersection indicate the number of patients positive for the different combinations of autoantibodies.

| **A.** |  |
| --- | --- |
| 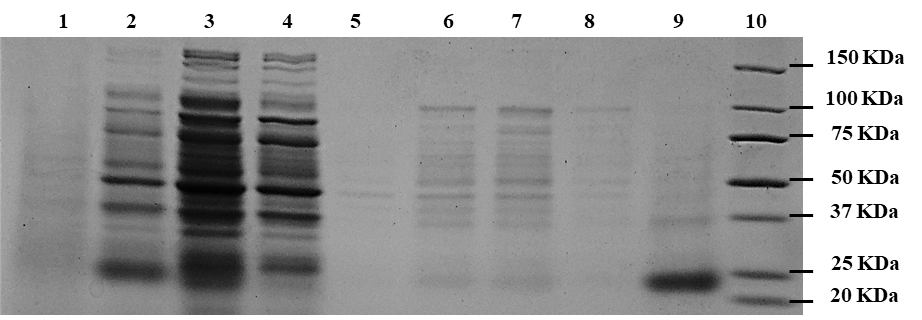 |  |
| **B.** |  |
| 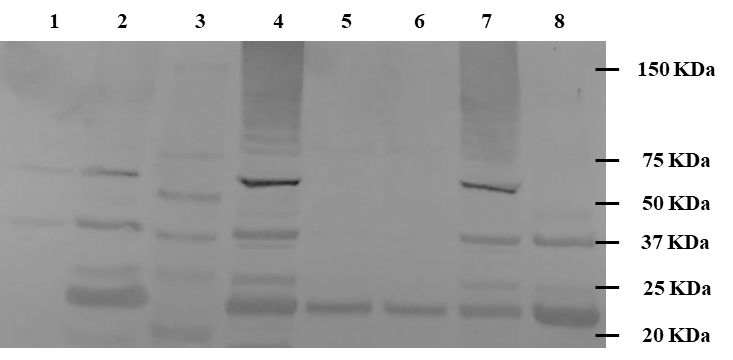 |  |
| **Supplementary Figure 2: Analysis of purification of TrxPI from ISF by affinity chromatography and isolation from IB, by SDS-PAGE (A) and Western blotting (B)**. In **(A)**, lane 1 corresponds to total lysate of transformed *E. coli* strain GI724 before induction, lane 2: total lysate of transformed *E. coli* strain GI724 after induction, lane 3: total ISF, lane 4: unbound material, lane 5: wash step with 2ME 5 mM, lanes 6 and 7: consecutive eluates of purified TrxPI, lane 8: TrxPI biotinylated, lane 9: TrxPI from IB, lane 10: Molecular Weight Markers. In **(B)**, lane 1 corresponds to total lysate of transformed *E. coli* strain GI724 before induction, lane 2: total lysate of transformed *E. coli* strain GI724 after induction, lane 3: Molecular Weight Markers, lane 4: total ISF, lane 5: purified TrxPI, lane 6: TrxPI biotinylated, lane 7: unbound material, lane 8: TrxPI from IB. | |

**Supplementary table**

**Supplementary Table 1: Raw data from 100 samples from normal human controls and from 111 type 1 diabetic patients analyzed by FloCMIA.** GeoM values are reported in duplicate for each sample and the corresponding Standard Deviation score (SDs) value.

| **Normal human controls** | **GeoM 1** | **GeoM 2** | **SDs** |
| --- | --- | --- | --- |
| 1^*^ | 6.98 | 8.96 | 0.028 |
| 2^*^ | 13.00 | 11.56 | 2.452 |
| 3^*^ | 7.20 | 7.80 | -0.236 |
| 4^*^ | 9.40 | 9.40 | 0.832 |
| 5^*^ | 6.50 | 6.96 | -0.669 |
| 6^*^ | 9.30 | 8.82 | 0.641 |
| 7^*^ | 7.30 | 7.78 | -0.213 |
| 8^*^ | 7.20 | 7.08 | -0.438 |
| 9^*^ | 6.98 | 7.02 | -0.517 |
| 10^*^ | 7.01 | 8.17 | -0.185 |
| 11^*^ | 6.50 | 6.90 | -0.686 |
| 12^*^ | 7.23 | 7.31 | -0.365 |
| 13^*^ | 6.45 | 6.93 | -0.691 |
| 14^*^ | 7.15 | 7.97 | -0.202 |
| 15^*^ | 12.29 | 12.41 | 2.491 |
| 16^*^ | 6.37 | 6.99 | -0.697 |
| 17^*^ | 6.22 | 6.42 | -0.899 |
| 18^*^ | 9.14 | 9.88 | 0.894 |
| 19^*^ | 5.70 | 6.18 | -1.113 |
| 20^*^ | 7.32 | 7.00 | -0.427 |
| 21^*^ | 5.43 | 5.43 | -1.102 |
| 22^*^ | 6.00 | 6.84 | -0.356 |
| 23^*^ | 5.99 | 6.29 | -0.567 |
| 24^*^ | 6.11 | 6.37 | -0.492 |
| 25^*^ | 7.42 | 7.48 | 0.420 |
| 26^*^ | 7.22 | 7.50 | 0.352 |
| 27^*^ | 5.88 | 6.62 | -0.484 |
| 28^*^ | 11.00 | 10.08 | 2.748 |
| 29^*^ | 42.23 | 48.27 | 20.989 |
| 30^*^ | 18.99 | 21.43 | 10.258 |
| 31 | 2.690 | 2.370 | 1.139 |
| 32 | 1.820 | 1.720 | -0.177 |
| 33 | 1.840 | 1.620 | -0.246 |
| 34 | 1.980 | 1.990 | 0.195 |
| 35 | 1.990 | 1.680 | -0.064 |
| 36 | 1.550 | 1.510 | -0.593 |
| 37 | 1.490 | 1.530 | -0.627 |
| 38 | 1.590 | 1.550 | -0.523 |
| 39 | 1.600 | 1.570 | -0.497 |
| 40 | 2.080 | 2.470 | 0.698 |
| 41 | 1.830 | 1.750 | -0.142 |
| 42 | 1.570 | 1.620 | -0.480 |
| 43 | 1.730 | 1.720 | -0.255 |
| 44 | 1.510 | 1.490 | -0.645 |
| 45 | 1.550 | 1.550 | -0.558 |
| 46 | 4.500 | 3.380 | 3.581 |
| 47 | 1.610 | 1.630 | -0.437 |
| 48 | 4.220 | 2.200 | 2.317 |
| 49 | 1.490 | 1.530 | -0.627 |
| 50 | 1.600 | 1.580 | -0.489 |
| 51 | 2.300 | 1.610 | 0.143 |
| 52 | 1.590 | 1.630 | -0.454 |
| 53 | 1.750 | 1.580 | -0.359 |
| 54 | 1.670 | 1.600 | -0.411 |
| 55 | 1.580 | 1.600 | -0.489 |
| 56 | 1.600 | 1.630 | -0.555 |
| 57 | 1.890 | 1.890 | -0.053 |
| 58 | 1.630 | 5.020 | 2.743 |
| 59 | 1.620 | 1.620 | -0.545 |
| 60 | 1.710 | 1.900 | -0.188 |
| 61 | 3.000 | 3.750 | 2.840 |
| 62 | 1.630 | 1.600 | -0.555 |
| 63 | 1.770 | 1.700 | -0.323 |
| 64 | 1.640 | 1.760 | -0.391 |
| 65 | 1.580 | 1.550 | -0.651 |
| 66 | 1.620 | 1.640 | -0.526 |
| 67 | 1.610 | 1.800 | -0.381 |
| 68 | 2.790 | 2.140 | 1.085 |
| 69 | 1.820 | 1.680 | -0.294 |
| 70 | 1.980 | 1.850 | 0.024 |
| 71 | 1.640 | 1.640 | -0.506 |
| 72 | 1.670 | 2.080 | -0.053 |
| 73 | 1.740 | 1.620 | -0.429 |
| 74 | 1.980 | 1.710 | -0.111 |
| 75 | 1.630 | 1.580 | -0.574 |
| 76 | 1.600 | 1.630 | -0.555 |
| 77 | 125.31 | 154.59 | 266.244 |
| 78 | 3.07 | 1.66 | 2.640 |
| 79 | 1.64 | 1.67 | -0.375 |
| 80 | 1.65 | 1.63 | -0.439 |
| 81 | 1.69 | 1.64 | -0.332 |
| 82 | 2.15 | 1.80 | 0.984 |
| 83 | 1.67 | 1.82 | 0.007 |
| 84 | 1.62 | 1.60 | -0.566 |
| 85 | 1.60 | 1.66 | -0.481 |
| 86 | 1.73 | 1.66 | -0.205 |
| 87 | 1.79 | 1.67 | -0.056 |
| 88 | 1.68 | 1.69 | -0.247 |
| 89 | 1.88 | 1.92 | 0.666 |
| 90 | 1.75 | 1.61 | -0.269 |
| 91 | 1.78 | 1.76 | 0.114 |
| 92 | 1.71 | 1.78 | 0.007 |
| 93 | 1.65 | 1.58 | -0.545 |
| 94 | 1.62 | 1.62 | -0.523 |
| 95 | 1.64 | 1.69 | -0.332 |
| 96 | 2.00 | 1.95 | 0.840 |
| 97 | 1.73 | 1.80 | 0.092 |
| 98 | 1.66 | 1.61 | -0.460 |
| 99 | 1.63 | 1.63 | -0.481 |
| 100 | 1.71 | 1.72 | -0.184 |
| **Type 1 diabetic patients** | **GeoM 1** | **GeoM 2** | **SDs** |
| 1^*^ | 32.83 | 34.81 | 14.562 |
| 2^*^ | 41.04 | 38.72 | 17.970 |
| 3^*^ | 12.12 | 12.18 | 3.880 |
| 4^*^ | 8.05 | 8.05 | 0.073 |
| 5^*^ | 6.14 | 8.22 | -0.146 |
| 6^*^ | 6.99 | 8.23 | -0.174 |
| 7^*^ | 18.20 | 18.28 | 5.803 |
| 8^*^ | 18.75 | 19.57 | 9.241 |
| 9^*^ | 14.26 | 16.94 | 4.318 |
| 10^*^ | 5.08 | 6.80 | -1.113 |
| 11^*^ | 6.90 | 7.00 | -0.545 |
| 12^*^ | 18.20 | 22.22 | 10.032 |
| 13^*^ | 7.65 | 7.77 | -0.118 |
| 14^*^ | 9.18 | 9.16 | 1.716 |
| 15^*^ | 6.89 | 7.61 | 0.269 |
| 16^*^ | 13.82 | 15.82 | 3.880 |
| 17 | 55.55 | 39.52 | 88.009 |
| 18 | 2.760 | 5.220 | 4.026 |
| 19 | 134.27 | 155.51 | 275.772 |
| 20 | 145.910 | 154.470 | 285.993 |
| 21 | 3.32 | 6.97 | 6.253 |
| 22 | 703.77 | 761.38 | 1265.314 |
| 23 | 340.900 | 239.640 | 556.158 |
| 24 | 110.64 | 120.32 | 483.031 |
| 25 | 405.05 | 432.97 | 722.332 |
| 26 | 9.400 | 4.280 | 9.522 |
| 27 | 141.15 | 137.89 | 240.189 |
| 28 | 285.81 | 357.21 | 553.497 |
| 29 | 1.7 | 1.7 | -0.298 |
| 30 | 8.18 | 4.31 | 8.375 |
| 31 | 8.78 | 9.06 | 13.534 |
| 32 | 484.13 | 472.41 | 824.949 |
| 33 | 72.340 | 46.150 | 110.593 |
| 34 | 3.820 | 2.280 | 2.213 |
| 35 | 9.02 | 3.63 | 8.529 |
| 36 | 336.560 | 229.460 | 542.156 |
| 37 | 109.830 | 81.240 | 180.583 |
| 38 | 292.780 | 451.850 | 714.393 |
| 39 | 560.700 | 530.310 | 1048.415 |
| 40 | 201.25 | 80.92 | 241.066 |
| 41 | 2.040 | 1.870 | 0.101 |
| 42 | 187.22 | 275.87 | 397.710 |
| 43 | 4.58 | 4.19 | 4.351 |
| 44 | 145.73 | 91.77 | 202.390 |
| 45 | 2.24 | 2.44 | 0.810 |
| 46 | 2.27 | 2.04 | 0.490 |
| 47 | 225.87 | 188.41 | 355.450 |
| 48 | 150.17 | 142.78 | 278.828 |
| 49 | 1.81 | 1.65 | -0.246 |
| 50 | 4.64 | 3.99 | 4.653 |
| 51 | 114.93 | 75.68 | 161.792 |
| 52 | 1.68 | 1.67 | -0.341 |
| 53 | 3.17 | 3.23 | 1.433 |
| 54 | 5.45 | 4.63 | 5.485 |
| 55 | 1.68 | 1.6 | -0.402 |
| 56 | 119.19 | 129.47 | 467.383 |
| 57 | 1.63 | 1.57 | -0.808 |
| 58 | 1.55 | 1.57 | -0.961 |
| 59 | 1.66 | 1.63 | -0.637 |
| 60 | 1.62 | 1.62 | -0.732 |
| 61 | 1.63 | 1.66 | -0.637 |
| 62 | 204.16 | 256.32 | 877.131 |
| 63 | 3.57 | 2.49 | 4.647 |
| 64 | 9.77 | 7.61 | 26.239 |
| 65 | 2.56 | 2.64 | 3.007 |
| 66 | 2.61 | 2.5 | 2.835 |
| 67 | 2.53 | 2.45 | 2.587 |
| 68 | 2.77 | 2.57 | 3.274 |
| 69 | 2.48 | 2.51 | 2.606 |
| 70 | 4.87 | 4.86 | 11.647 |
| 71 | 94.8 | 9.81 | 192.621 |
| 72 | 398.64 | 313.16 | 1350.777 |
| 73 | 434.6 | 426.81 | 1636.143 |
| 74 | 13.23 | 5.93 | 29.634 |
| 75 | 2.6 | 2.5 | 2.816 |
| 76 | 2.56 | 2.73 | 3.178 |
| 77 | 6.86 | 4.44 | 14.642 |
| 78 | 5.03 | 2.88 | 8.176 |
| 79 | 265.49 | 181.38 | 845.449 |
| 80 | 30.03 | 55.28 | 155.809 |
| 81 | 1.6 | 1.59 | -0.827 |
| 82 | 1.98 | 2.09 | 0.851 |
| 83 | 1.63 | 1.64 | -0.675 |
| 84 | 1.6 | 1.55 | -0.904 |
| 85 | 1.59 | 1.58 | -0.865 |
| 86 | 1.67 | 1.6 | -0.675 |
| 87 | 1.53 | 1.61 | -0.923 |
| 88 | 1.6 | 1.63 | -0.545 |
| 89 | 1.73 | 1.73 | -0.056 |
| 90 | 1.78 | 1.62 | -0.184 |
| 91 | 1.65 | 1.59 | -0.523 |
| 92 | 1.59 | 1.87 | -0.056 |
| 93 | 1.58 | 1.62 | -0.608 |
| 94 | 1.58 | 1.64 | -0.566 |
| 95 | 1.61 | 1.56 | -0.672 |
| 96 | 447.63 | 333.1 | 1650.445 |
| 97 | 1.69 | 1.65 | -0.311 |
| 98 | 1.58 | 1.55 | -0.757 |
| 99 | 1.58 | 1.58 | -0.693 |
| 100 | 1.68 | 1.69 | -0.247 |
| 111 | 1.69 | 1.63 | -0.354 |

^*^Samples acquired on a PAS III PARTEC flow cytometer (PARTEC. Görlitz. Germany). The rest of the samples were acquired in a Becton Dickinson FACSCalibur flow cytometer (Franklin Lakes. NJ. USA).
